# Thermally Resistant, Self-Extinguishing Thermoplastic Composites Enabled by Tannin-Based Carbonaceous Particulate

**DOI:** 10.3390/polym14183743

**Published:** 2022-09-07

**Authors:** André L. Missio, Rafael A. Delucis, Caio Gomide Otoni, Pedro H. G. de Cademartori, Rodrigo Coldebella, Arthur B. Aramburu, Bruno D. Mattos, Marlon B. B. Rodrigues, Nayara Lunkes, Darci A. Gatto, Jalel Labidi

**Affiliations:** 1 Graduate Program in Materials Science and Engineering (PPGCEM), Technology Development Center, Federal University of Pelotas (UFPel), Pelotas 96010-610, RS, Brazil; 2 Graduate Program in Environmental Sciences (PPGCAmb), Engineering Center, Federal University of Pelotas (UFPel), Pelotas 96010-610, RS, Brazil; 3 Department of Materials Engineering (DEMa), Federal University of São Carlos (UFSCar), São Carlos 13565-905, SP, Brazil; 4 Graduate Program in Forestry Engineering (PPGEF), Federal University of Paraná, Curitiba 80210-170, PR, Brazil; 5 Forest Products Laboratory (PPGEF), Center for Rural Sciences, Federal University of Santa Maria (UFSM), Santa Maria 97105-900, RS, Brazil; 6 Department of Bioproducts and Biosystems, School of Chemical Engineering, Aalto University, FI-00076 Espoo, Finland; 7 Materials Engineering Course, Technological Development Center, Federal University of Pelotas (UFPel), Pelotas 96010-610, RS, Brazil; 8 Architecture and Urbanism Undergraduate Course, Catholic University of Pelotas (UCPel), Pelotas 96015-560, RS, Brazil; 9 Chemical and Environmental Engineering Department, University of the Basque Country, Escuela Politécnica de San Sebastián, Plaza Europa, 1, 20018 Donostia-San Sebastián, Guipuzcoa, Spain

**Keywords:** bioeconomy, tannin foams, engineering components, building materials, flame-resistant materials, polypropylene

## Abstract

Flame-resistant materials are key components in buildings and several other engineering applications. In this study, flame retardancy and thermal stability were conferred to a highly flammable technical thermoplastic—polypropylene (PP)—upon compositing with a carbonaceous tannin-based particulate (CTP). Herein, we report on a straightforward, facile, and green approach to prepare self-extinguishing thermoplastic composites by thermoblending highly recalcitrant particulate. The thermal stability and mechanical properties of the composites are tethered to the CTP content. We demonstrate that the addition of up to 65 wt% of CTP improved the viscoelastic properties and hydrophobicity of the PP, whereas having marginal effects on bulk water interactions. Most importantly, compositing with CTP remarkably improved the thermal stability of the composites, especially over 300 °C, which is an important threshold associated with the combustion of volatiles. PP-CTP composites demonstrated great capacity to limit and stop fire propagation. Therefore, we offer an innovative route towards thermally resistant and self-extinguishing PP composites, which is enabled by sustainable tannin-based flame retardants capable of further broadening the technical range of commodity polyolefins to high temperature scenarios.

## 1. Introduction

Plastics play a crucial role in many branches of engineering, spanning from construction to textiles. Polyolefins, including polypropylene (PP), are commodity plastics that find a plethora of general purpose uses owing to their easy processing and recyclability, high chemical resistance, and low cost [1,2]; however, their hydrocarbon nature and relatively low melting point (ca. 130–170 °C for PP, depending mainly on tacticity) make them unsuitable for applications involving moderate to high temperatures, such as in the automotive, aerospace, and building segments [3,4,5]. Additionally, the olefinic structure of PP dissolves into highly volatile aromatic hydrocarbons (e.g., toluene and benzene) under thermal stresses (slightly above 100 °C) and can easily ignite by either heat or flame, releasing poisonous gases upon burning. Therefore, several attempts of rendering PP or its composites (e.g., fiber-reinforced PP) flame retardant or even self-extinguishing have been made over the last years [6,7,8,9,10,11,12].

Flame retardancy in PP and other flammable materials is often achieved with the introduction of additives, which can either act as recalcitrant components that burn at a slow rate and are less likely to ignite or by quenching chain reactions during combustion. In the past, few substances based on chlorine and bromine were used to improve both thermal and combustion resistance in PP [5,13]. More recently, few halogen-free flame-retardant systems, which are divided into additives or reactive substances [5], have been proven to be highly efficient. The latter includes compounds based on metal hydroxides, metal borates, and intumescent flame retardants [4,13,14,15]. As noted, the compounds used so far are heavily dependent on highly energy demanding mining or synthesis. Although synthetic flame retardants provide high thermal and/or combustion resistance, their non-renewable nature in parallel to their often poor interface with most polymer matrices [16] has incentivized the search for bio-based components that can fulfil flame retardancy and other requirements while adding sustainability to such materials.

Natural polyphenolics, such as lignins and tannins, have been demonstrated to improve the thermal stability of PP composites [17]. Their recalcitrant, aromatic nature and their ability to char under thermal stress have been key for their utilization as thermally stable fillers [18]. Flame retardancy, however, has not been achieved using only natural polyphenolics. For such endeavors, natural polyphenolics have been modified with more traditional, inorganic compounds via supramolecular [19] or covalent [20] interactions to be later incorporated into PP matrices. In this context, we herein demonstrate the use of carbonaceous particulate composed of tannin-furfuryl alcohol copolymers (CTP) to produce and thermally resistant, self-extinguishing PP composites. The combination of tannins, especially their condensed structures, with furfuryl alcohol, another bio-based compound, has been widely investigated recently for the formation of insulating and flame-retardant foams by their cross-linking reaction with aldehydes [21,22]. Such foams, however, are highly brittle, thus limiting applications where flexibility and/or ductility are required [23,24,25,26]. Therefore, we composited a CTP made up of tannin and furfuryl alcohol as a bio-based flame retardant with PP as matrix to reach balanced fire safety as well as thermal and mechanical properties, thus fully harnessing the advantages of each individual component while making more sustainable flame-retardant materials.

## 2. Materials and Methods

### 2.1. Raw Materials

Condensed tannins were extracted from black wattle bark (*Acacia mearnsii*) and supplied by TANAC^®^ (Montenegro, Brazil). According to the supplier, this tannin extract is composed of condensed tannins (70–80 wt%), hydrocolloid gums (20–30 wt%), as well as minor amounts of sugars and small molecules. Its particle size mean was determined by scanning electron microscopy (SEM) to be 0.66 μm. Neat PP (H103) was supplied by Braskem (Triunfo, Brazil). According to the supplier, this polymer matrix has the following characteristics: 40 g·min^−1^ melt flow rate at 230 °C/2.16 kg (ASTM D1238), 0.905 g·cm^−3^ density (ASTM D792), 1200 MPa bending modulus (ASTM D790), 34 MPa tensile strength (ASTM D638), 101 Rockwell hardness (ASTM D785), 20 J/m Notched Izod strength (ASTM D256), and 156 °C Vicat softening point (ASTM D1525). The following chemicals were acquired from Sigma Aldrich, Guarulhos, Brazil, and used without previous purification: furfuryl alcohol (CAS number 98-00-0), formaldehyde (CAS number 50-00-0), diethyl ether (CAS number 60-29-7), and toluene-4-sulfonic acid (CAS number 6192-52-5).

The CTP derived from rigid tannin foams was produced accordingly with Tondi and Pizzi [23]. *A. mearnsii* tannin extract, distilled water, furfuryl alcohol (97 wt%), and formaldehyde (32 wt%) were mixed under mechanical stirring for 2 min and added by diethyl ether (99.5 wt%) and toluene-4-sulfonic acid (65 wt%), followed by mechanical stirring for 30 s before the homogeneous mixture was then poured into an open container for the foam to rise. The foams were allowed to rest at room temperature (about 20 °C) for at least 24 h to evaporate unreacted volatiles and then knife-milled into CTP.

### 2.2. Preparation of the PP-CTP Composites

PP and CTP were mixed at four PP contents ranging from 35 to 65 wt% on a high-speed thermo-kinetic mixer (model MH-100, Guarulhos, Brazil) set at 120 °C (the temperature was further risen by viscous dissipation upon shearing) and then compression molded on an electrically heated hydraulic press (Marconi, model MA 098/AR15, Piracicaba, Brazil) at 900 MPa and 175 °C for 10 min. The molded parts (140 × 140 × 3.5 mm^3^) were produced in triplicates and equilibrated at 20 °C and 65% relative humidity in an environmental chamber until reaching constant mass.

### 2.3. Fourier-Transformed Infrared (FTIR) Spectroscopy

The chemical features of the samples were investigated by FTIR. KBr pellets were prepared with milled samples and then analyzed in direct transmittance mode on an infrared spectrometer (Shimadzu Prestige-21, Kyoto, Japan). A total of 45 scans were recorded at a resolution of 2 cm^−1^ in the 400–4500 cm^−1^ wavenumber range. The spectra were normalized (0, 1) to enable intensity comparisons.

### 2.4. Density and Bulk Water Interactions

Apparent density (ASTM D792), water absorption, and thickness swelling at 2 and 24 h (ASTM D570) were determined for all composites following their respective standardized procedures. Water leaching was determined in triplicate: 1 g of a milled sample (60 mesh) was immersed in distilled water for 24 h and then oven dried at 60 °C until reaching constant mass. Quantitative data were statistically analyzed using analysis of variance prior to Tukey tests, as suitable, both at a significance level of 5%.

### 2.5. Scanning Electron Microscopy

The burnt section of the PP-CTP composite was gold-sputtered and imaged on a scanning electron microscope (Phenom ProX Desktop SEM, Guarulhos, Brazil) using a 5 kV voltage.

### 2.6. Surface Wettability

Water contact angle was measured through the sessile drop method on a goniometer (Kruss, model DSA25B, Hamburg, Germany). Distilled water drops (11 µL in volume) were cast on the surface of five samples (20 × 20 mm^2^) per treatment and the contact angle was measured every 10 s for 60 s.

### 2.7. Thermogravimetry

The mass loss profile over temperature was evidenced by thermogravimetric (TG) and derivative TG (DTG) curves recorded from 30 to 600 °C at a constant heating rate of 15 °C·min^−1^, within an argon flow rate of 50 mL·min^−1^.

### 2.8. Differential Scanning Calorimetry (DSC)

Differential scanning calorimetry (DSC) was performed using a TA Q20 calorimeter (New Castle, DE, USA) in the 100–200 °C range in order to evaluate temperatures of crystallization (Tc) and melting (Tm) of CTP-PP composites. The samples were heated, cooled down, and then heated again, always at a rate of 10 °C·min^−1^ in the ramp mode. Crystallinity (%) was calculated based on the area under curve, according to Mattos et al. [27].

### 2.9. Dynamic Mechanical Analysis and Tensile Tests

Mechanical behavior of the PP-CTP composites was studied on a dynamic mechanical analyzer (TA instruments, model Q-800, New Castle, DE, USA). Prismatic (35 × 17 × 37 mm^3^) specimens were evaluated using a dual cantilever set up at a frequency of 1 Hz and a static load of 5 N. Samples were heated from 30 to 120 °C at 5 °C·min^−1^. Tensile properties of the PP-CTP composites were evaluated through mechanical tests using an EMIC DL23-300 universal machine (São José dos Pinhais, Brazil). The samples and test parameters were adjusted as indicated by ASTM D638. The samples had dimensions of 85 × 15 × 3 mm^3^ (length, width and thickness) and 5 replicates were used per treatment. The test speed was 5 mm·min^−1^.

### 2.10. Flame Retardancy

Flammability tests were performed to investigate the potential of CTP to act as a flame retardant in PP-based composites. Prismatic (15 × 3 × 100 mm^3^) specimens were exposed to a vertical flame from a Bunsen burner for 10 s, and then classified according to their visual aspect into totally burned (a) and self-extinguishing (b). The final weight loss was also measured. This test was inspired in an appropriated standard procedure. Pine wood sawdust- and pine needle-filled PP composites (50 wt% PP), prepared following the same conditions described earlier, were used as a comparison. Unfilled neat PP specimens were also tested for comparison purposes.

### 2.11. Statistical Analyses

Data normality was confirmed by Shapiro-Wilk tests. Afterwards, all the data were subjected to ANOVA tests followed by Fisher tests. The latter tests were performed to compare the means. All statistical tests were conducted at a 0.05 significance level.

## 3. Results and Discussion

### 3.1. Chemical Features of the Composites

**Figure 1:**
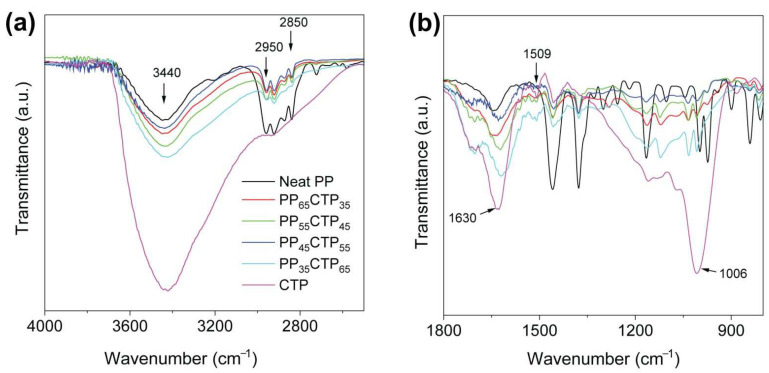
Infrared spectra for the PP−CTP composites and their single phases ranging from (**a**) 4000 to 2400 cm^−1^ and from (**b**) 1800 to 800 cm^−1^.

The FTIR spectra (Figure 1) of the composites display the typical spectroscopic signatures of their components. Neat PP has a prominent band at the 2850–2950 cm^−1^ range, which represents CH, CH_2_, and CH_3_ groups [27]. The broad peak at 3440 cm^−1^ is ascribed to the stretching vibration of the O–H groups associated with absorbed moisture [28], which is more intense for the CTP when compared to either neat PP or its composites. There is a positive correlation between the band centered at 3440 cm^−1^ and the CTP content in the composites, thus inferring a gradual decrease in hydrophilicity associated with a progressive increase in PP fraction in the composite, which is expected because of the hydrocarbon, nonpolar nature of PP.

The IR spectrum of CTP had peaks at 1006, 1509, and 1630 cm^−1^. The peak at 1006 cm^−1^ is attributed to C–O vibrations of (i) –C–O–C– ether bridges in complex oxygen-containing cyclic molecules and/or (ii) =C–O–C= breathing in furan moieties [28]. That band at 1509 cm^−1^ can be associated with in-plane C–H bending vibrations in aromatic molecules from heteroaromatic compounds [28]. This band can also be related to C=C stretching vibrations in furan rings belong to the furfuryl alcohol [29,30]. The band at 1630 cm^−1^ is typically attributed to C=C stretching in benzene ring. As noted, there were no new clear IR peaks in the composite spectra that would indicate the formation of new chemical identities. Therefore, the PP-CTP interfacial interactions are driven primarily by physical means, i.e., Van der Waals contact forces including London dispersion. Hydrophobic interactions and hydrogen bonding may also be at play.

### 3.2. Density and Water Interactions

**Figure 2:**
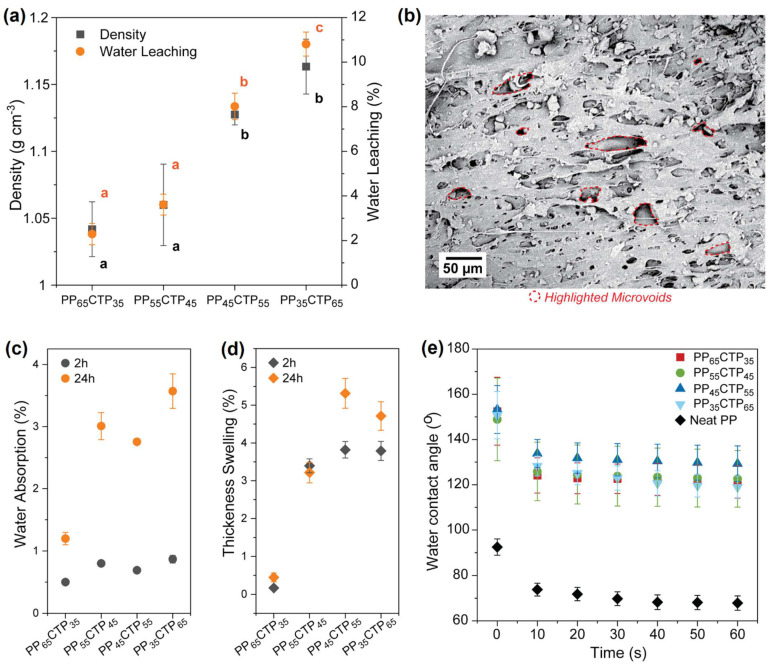
Water-composite relationships. (**a**) Density and water leaching of the PP-CTP composites comprising CTP at 35 (PP_65_CTP_35_) to 65 wt% (PP_35_CTP_65_). (**b**) Scanning electron microscopy of the PP_55_CTP_45_ composite, displaying the presence of microvoids across its structure. (**c**) Water absorption and (**d**) thickness swelling (TS) after 2 and 24 h. (**e**) Water contact angle kinetics for the PP-CTP composites. Values having the same letter (**a**) are not significantly different (Fisher LSD test).

The addition of the CTP into the PP matrix increased the apparent density of the composites in a quasi-linear fashion (Figure 2). By gravimetry, we have estimated the density of the CTP to be ca. 1.5 g·cm^−3^, which is much higher than the values obtained for classic tannin-rigid foams—ranging from 0.05 to 0.18 g·cm^−3^ [24,31]. The density of the composites did not follow the rule of mixtures as a result of the formation of microvoids during the composite processing. These microvoids can be ascribed to the weak interfacial adhesion already expected for the studied phases. Similar increases in density were reported for PP-based composites filled with other bio-based materials, such as wood particles [17] and starch [32]. An increase in the density is majorly attributed to the density of the filler, even when chemical treatments are applied to the filler or small amounts of additives are incorporated into the matrix [32].

Water leaching is usually associated with water resistance in PP-based composites [15] and it is crucial to the implementation of this composite in several applications involving liquid media, at least potentially [14]. There is a positive relationship between CTP content and water leaching (Figure 2a). The multiphase (not fully compacted) character of the composite, as well as the presence of microvoids at the components interface (Figure 2b), allow a partial water flow in and out of the material in some conditions (e.g., full immersion). Given the heterogeneous nature of CTP, solubilization of some entities may be inducing the leaching of relatively bigger particles. However, even at such harsh conditions, only ca. 10 wt% of the material is lost after 24 h. This indirectly confirms the physically driven PP-CTP interaction, corroborating FTIR. Nevertheless, most of the traditional flame retardants (both additives and intumescent flame retardants) are easily leached in water if no strong interactions with the matrix are in place, as these are based on amines, phosphates, and sodium salts, among others [5,14].

Neat PP does not absorb water. Considering the hydrophilic character of CTP, one may expect an increase in the water absorption and thickness swelling of the PP composites compared to neat PP (Figure 2c,d). This stronger interaction with water agrees with most of the PP-based composites, and it is tethered to the higher polarity of chemical groups belonging to the filler, i.e., groups able to H-bond with water such as hydroxyls [17,32]. Although higher water absorption is found in all the composites when compared to neat PP, it is remarkable that the values were below 5% in all conditions, especially when no covalent bonds are formed between the composite components. The overall increase in water absorption and thickness swelling, although not significant, probably resulted from the appearance of microvoids at the PP/filler interface (Figure 2b). Furthermore, the SEM images seem to indicate an overall good distribution of the filler particles inside the polymer matrix.

As far as surface wetting, the addition of CTP resulted in composites with remarkably higher water contact angle when compared to neat PP (Figure 2e), reaching a difference of 50° in the best performers. One can note that bulk and surface water relationship remarkably differ from each other in our materials. Kaymakci and Ayrilmis [33] incorporated variable wood flour contents (ranging from 30 to 60 wt%) into a PP matrix and reported that the increases in wood flour content yielded increased surface wettabilities, which were attributed to increases in surface roughness as smooth surfaces may facilitate drop spreading while air pockets may prevent it. Processing temperature and viscosity also strongly influence the final surface wettability of typical bio-based PP composites [34]. The contact angle kinetics also indirectly indicates that the CTP were homogeneously distributed inside the PP-based matrix as all composites presented similar surface energies. This substantial increase in the surface hydrophobicity of PP-CTP composites can also be assigned to the nonpolar nature of some of the tannin moieties that are associated to aromatic groups. Whereas more hydrophilic substrates may improve bonding strength and adhesion, a more hydrophobic surface is key for outdoor applications as a higher water contact angle usually indicates better resistance against biologic degradation (e.g., by insects and fungi) [17].

### 3.3. Thermal, Mechanical and Thermo-Mechanical Properties of the Composites

**Figure 3:**
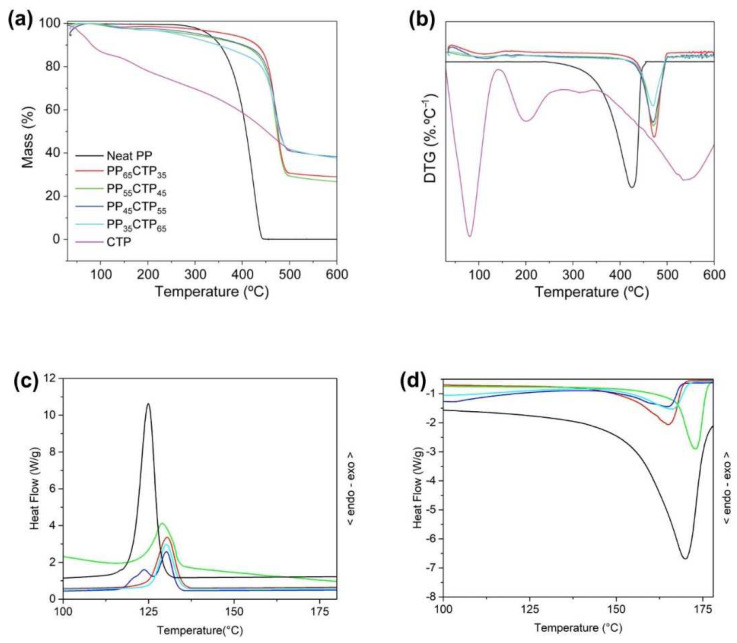
(**a**) Thermogravimetric (TG) and (**b**) derivative TG (DTG) curves, and DSC curves for (**c**) first and (**d**) second heatings for the PP-CTP composites and their single phases.

All composites presented similar thermal decomposition profiles (Figure 3), which are not simple superimposition of the thermograms of their single phases, especially until 400 °C. This behavior is different from what was reported in most studies on bio-filled PP composites, which affirmed that the thermal stability followed closely the rule of mixtures [1,8,17]. Chemical interactions between filler and matrix do not exist or are in too low intensity that could not be observed by FTIR. Therefore, the remarkable difference between the thermal behavior of the tannin-furan based powder outside and inside the PP matrix can be attributed to the accessible surface area and heat exchange kinetics. The particulate material exchanges heat with the environment in a faster way than their bulkier counterparts. This has been studied in the case of glass transition temperature of microparticles when compared to their nanoscaled analogues [35,36].

The neat PP was more thermally stable than the single CTP up to ~400 °C, which is the range where the neat PP starts its thermal decomposition. PP completely degrades after 450 °C following a series of random chain scissions and chain transfer reactions, releasing pentane and heptane, until complete decomposition [4,7,37]. On the other hand, the CTP is a multi-branched rigid copolymer composed by aromatic moieties from their tannin-furan networks that can rearrange themselves into more compacted carbonized structures during thermal stresses [24,38,39].

The CTP incorporated in the PP matrix shifted the maximum decomposition peak, which is ascribed to PP, to ca. 470 °C, as well as increased its residual content to above 20 wt%. In buildings, whenever there is any risk for human life, this can help to extend the escape time during a conflagration. This behavior occurred for other PP-based composites and it is ascribed to the formation of char during the filler decomposition [37]. A high residue yield is also related to high flame resistance [37]. Still compared to the neat PP, the PP-CTP composites had similar thermal stabilities until 300 °C, at which point they became more thermally resistant. This is also an important finding because this temperature level is easily exceeded during a conflagration. The Tm and Tc results determined with basis on the DSC curves indicated that, in relation to the neat PP, small increases of 4–5 °C were found for all CTP-based composites. These increases were observed as a function of the CTP content. Therefore, the CTP particles probably acted as nucleating agents for the crystallization of PP [27]. This impacted the PP dispersion and promoted a heterogeneous nucleation [40]. The Tm results kept stable for all composites. As observed in Table 1, the ΔHf and Xc decreased with addition of the CTP particles. This decrease is related to the formation of a transcrystalline region, in which restrictions in the lateral direction of growth of spherulites are observed, resulting in a columnar layer [41]. Similar results were reported in previous studies on particulate filled PP composites [27,40].

**Figure 4:**
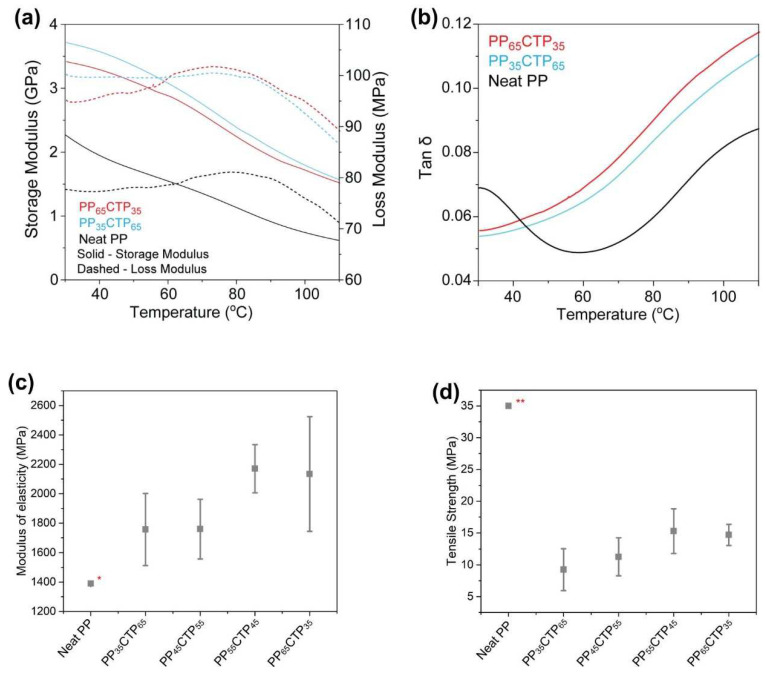
(**a**) Storage (E′) and loss (E″) moduli, (**b**) Tan δ curves for neat PP and PP-CTP composites, (**c**) modulus of elasticity (**d**) and tensile strength (MPa) for the PP-CTP composites. * and ** determined by Mattos et al. [27] and Sui et al. [40], respectively.

An important feature of materials aiming at either fire protection or thermal stability is their mechanical behavior as a function of temperature. Variations of storage (E′) and loss (E″) moduli were observed in the temperature range of 30–110 °C (Figure 4a). Compared to the neat PP, the composites presented both higher E′ and E″ across this temperature range, which indicates that the incorporation of the CTP led to remarkable gains in stiffness. Particles incorporated in soft matrices can create anchoring mechanisms among the polymer chains, thus decreasing their mobility and consequently stiffening the composite [17,37]. This is especially true when using porous reinforcements as, during processing, the PP melt can flow into the particulate pores, thus creating a mechanical interlocking that does not need to rely upon any chemical interactions [4,34].

All samples presented similar decreases in E′ when heated, which means similar softening and relaxation processes. The similarly shaped E″ kinetics indicates that the CTP insertion did not change the viscoelastic nature of the neat PP, which probably did not affect molecular rearrangement mechanisms during the loading cycles [37]. The relaxation transition peak at ca. 70 °C for the neat PP appeared similar to an inflection in the Tan δ curve (Figure 4b), probably representing the α-transition of PP crystalline fractions [39]. Both tensile strength (F = 3.61; *p* > 0.05) and tensile modulus (F = 3.12; *p* > 0.05) of the studied composites were considered statistically similar at a confidence level of 0.95%. Analog trends we observed in previous studies [2,17,27].

### 3.4. Flame Resistance of the Composites

**Figure 5:**
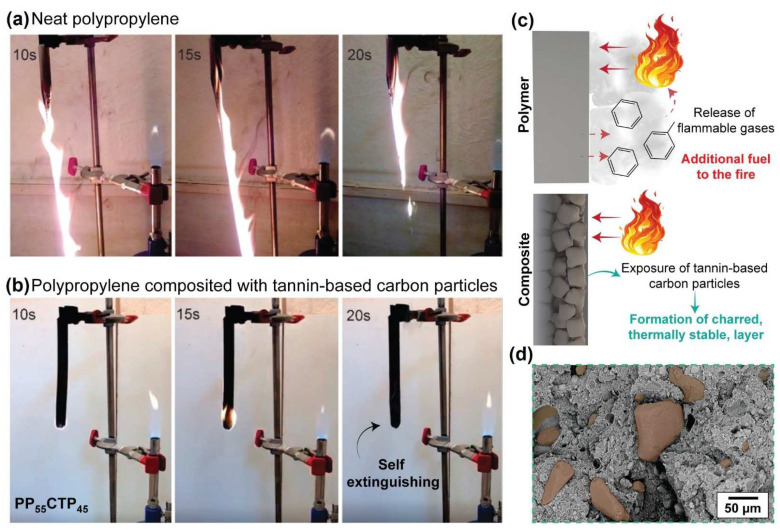
Behavior of (**a**) neat PP and (**a**,**b**) PP-CTP composite during combustion assays. (**c**) Proposed mechanism of flame development and burning process of the PP matrix and PP-CTP composites. (**d**) SEM image of the PP-CTP composite after burning displays the formation of the charred layer, and exposure of tannin microparticles (highlighted in brown) that induce flame extinguishing.

The burning tests showed that the neat PP and both positive controls (wood-polymer composites) underwent fast combustions and were fully degraded, as shown in Figure 5 and indicated in Table 2. Kim et al. [5] reported that thermoplastic composites mostly get entirely burnt out after 10 s of flame contact in vertical burning experiments. According to Ikram et al. [7], flammability in neat PP is attributed to its aliphatic hydrocarbon backbone that dissolves into highly volatile and flammable aromatic hydrocarbons (e.g., benzene). Regarding the positive controls, when there is a weak filler/matrix interface, these phases burn separately; while the matrix undergoes its normal quick burning, the filler acts as a heat conductor instead of as an insulator, which increases the overall flammability. Thus, incompatible, non-retardant filler poorly wetted in the matrix may lead to easier heat conduction, penetration, and oxygen admittance to the filler itself [34].

Remarkably, all PP-CTP composites self-extinguished before 20 s. Reduced flammability in a composite is usually attributed to a thermally stable filler [37]. The thermally decomposed filler becomes a char layer on the top surface of the composite and then acts as a steady barrier (Figure 5c), hindering the transfer of heat to the PP matrix from the radiant heat source (Figure 5), which in turn protects the underlying polymer [4,7]. Here, the addition of 65% CTP resulted in a self-extinguished composite before 20 s, losing only 5.58% in mass. This also suggests that small amounts of CTP endowed with a high specific surface area can also act as fire-resistant additives. Additionally, even expensive intumescent flame-retardant systems act following this same mechanism. According to Shao et al. [14], intumescent flame retardants generate multicellular swollen chars on the surface of the polymer accompanied by decomposition of the blowing agent, slowing down the heating and oxygen transfer, thus protecting the substrate from both radiant heat flux and flame. Future research may address thermogravimetric analysis coupled with mass spectrometry (TGA-MS) to fully explain this flame mechanism. 

## 4. Conclusions

The incorporation of CTP increased the density and induced a remarkably smaller surface wettability (water contact angle over 120°) in the PP matrix. The composites also presented higher water solubility, absorption, and swelling capacities than neat PP, behavior that was expected due to the hydrophilic nature of CTP. Unlike water resistance, the CTP content influenced positively the thermal, thermo-mechanical, and combustion properties. In fact, the CTP acted as a reinforcement component, rather than a filler, yielding higher dynamic mechanical properties regardless of the temperature within the tested range. The PP-CTP composites were more thermally stable than neat PP, especially above 300 °C, which is important for applications where there is a risk of fire. Self-extinguishing was one of the most attractive properties of these PP-CTP composites, which confirms CTP as an effective renewable alternative to avoid flame spread. The reduced flammability of PP granted by CTP arises from a charred layer of the latter on the surface of the former, which otherwise burns readily. CTP is demonstrated as an efficient bio-based flame retardant for flammable commodity polymers, herein showcased by PP, further extending their range of potential applications to those involving moderately high temperatures.

## Figures and Tables

**Table 1:** Crystallization (Tc) and melting (Tm) temperatures, heat of fusion (ΔHf) and degree of crystallinity (Xc) for the CTP-PP composites.

	Tc (°C)	Tm (°C)	ΔHf (J/g)	Xc (%)
Neat PP	125	168	82.6	43.5
PP_65_CTP_35_	130	164	17.8	14.4
PP_55_CTP_45_	129	170	16.4	13.8
PP_45_CTP_55_	130	165	12.6	14.7
PP_35_CTP_65_	130	166	13.6	15.9

**Table 2:** Flammability results for the neat PP, controls, and CTP-filled composites.

Sample	Mass Loss (%)	Burning Rating
Neat PP	100	Total burning
PP/pine needle	100	Total burning
PP/pine sawdust	100	Total burning
PP_65_CTP_35_	2.21	Self-extinguishing
PP_55_CTP_45_	2.12	Self-extinguishing
PP_45_CTP_55_	2.01	Self-extinguishing
PP_35_CTP_65_	5.58	Self-extinguishing

## Data Availability

The data presented in this study are available upon request from the corresponding author.
